# Neurological Basis for Eye Movements of the Blind

**DOI:** 10.1371/journal.pone.0056556

**Published:** 2013-02-18

**Authors:** Rosalyn M. Schneider, Matthew J. Thurtell, Sylvia Eisele, Norah Lincoff, Elisa Bala, R. John Leigh

**Affiliations:** 1 Veterans Affairs Medical Center, Case Western Reserve University, Cleveland, Ohio, United States of America; 2 Department of Ophthalmology & Visual Sciences, University of Iowa, Iowa City, Iowa, United States of America; 3 Neurology Service and Center for the Prevention and Treatment of Visual Loss, Veterans Affairs Medical Center, Iowa City, Iowa, United States of America; 4 Jacobs Neurological Institute, State University of New York, Buffalo, New York, United States of America; 5 Department of Ophthalmology, Cleveland Clinic Foundation, Cleveland, Ohio, United States of America; Barrow Neurological Institute, United States of America

## Abstract

When normal subjects fix their eyes upon a stationary target, their gaze is not perfectly still, due to small movements that prevent visual fading. Visual loss is known to cause greater instability of gaze, but reported comparisons with normal subjects using reliable measurement techniques are few. We measured binocular gaze using the magnetic search coil technique during attempted fixation (monocular or binocular viewing) of 4 individuals with childhood-onset of monocular visual loss, 2 individuals with late-onset monocular visual loss due to age-related macular degeneration, 2 individuals with bilateral visual loss, and 20 healthy control subjects. We also measured saccades to visual or somatosensory cues. We tested the hypothesis that gaze instability following visual impairment is caused by loss of inputs that normally optimize the performance of the neural network (integrator), which ensures both monocular and conjugate gaze stability. During binocular viewing, patients with early-onset monocular loss of vision showed greater instability of vertical gaze in the eye with visual loss and, to a lesser extent, in the normal eye, compared with control subjects. These vertical eye drifts were much more disjunctive than upward saccades. In individuals with late monocular visual loss, gaze stability was more similar to control subjects. Bilateral visual loss caused eye drifts that were larger than following monocular visual loss or in control subjects. Accurate saccades could be made to somatosensory cues by an individual with acquired blindness, but voluntary saccades were absent in an individual with congenital blindness. We conclude that the neural gaze-stabilizing network, which contains neurons with both binocular and monocular discharge preferences, is under adaptive visual control. Whereas monocular visual loss causes disjunctive gaze instability, binocular blindness causes both disjunctive and conjugate gaze instability (drifts and nystagmus). Inputs that bypass this neural network, such as projections to motoneurons for upward saccades, remain conjugate.

## Introduction

Eye movements evolved to serve vision [1]. During locomotion, vestibular and visual tracking mechanisms act to stabilize the retinal image [2]. However, even while stationary, during attempted fixation of an earth-fixed target, small eye movements (microsaccades, drift, and tremor) are generated to prevent sensory adaptation, which would cause fading of the visual percept [3], [4]. In species with a fovea (a specialized area of the retina providing high visual acuity), saccades redirect the line of sight (the angle of gaze) toward features of interest [5].There is abundant evidence that the performance of these eye movements, including the vestibulo-ocular reflex [6], is optimized by visual feedback [7]–[9]. Such plastic-adaptive properties of eye movements in response to visual feedback constitute a type of motor learning, for which the cerebellum plays an important role [10].

One important component of gaze control is referred to as the neural integrator for eye movements, because it integrates premotor signals for saccades, pursuit, and vestibular inputs, which are velocity coded, into signals encoding eye position [2], [11]. This ocular motor integrator has been shown to depend on a distributed network of neurons, which include the medial vestibular nucleus and adjacent nucleus prepositus hypoglossi in the medulla, the interstitial nucleus of Cajal in the midbrain, and the vestibular cerebellum [12], [13]. The cellular mechanism by which neurons in the nucleus prepositus hypoglossi generate an eye position signal, has been identified as a cholinergic-mediated sustained depolarization [14]. Studies have shown that neurons in nucleus prepositus hypoglossi not only encode conjugate eye position, but also contain units that specify the position of each eye [15].

Since vision is used to optimize eye movements, a question arises: What is the effect of loss of vision on the control of eye movements? Prior studies of individuals who have visual loss showed that, during attempted fixation, gaze is unstable due to eye drifts, which may be disjunctive or even in opposite directions [16]–[21]. Some of these ocular drifts are pendular, but others are unidirectional with corrective saccades, thereby giving rise to jerk nystagmus. Individuals who have monocular loss of vision show reduced stability of gaze in the affected eye due to small, low-frequency, irregular oscillations that are predominantly vertical [19], [22]–[28]. Loss of vision lays bare certain fundamental properties about the control of eye movements, distinguishing those properties that are primarily dependent on visual feedback from those that are largely determined by anatomical pathways. In this sense, the consequences of visual loss upon the neural control of eye movements provide a window through which we can investigate one aspect of “nature versus nurture” in the human brain.

Unfortunately, many prior studies of eye movements in individuals who have visual impairment suffer from a methodological flaw: the calibration of eye movement measurements was either absent or unreliable. With only a few exceptions [19], [29], [30], prior studies have used electro-oculography, reflection-based, or video-based methods for measuring eye movements. When these methods are used to measure eye movements, calibration depends upon the subject’s ability to point the fovea (corresponding to the line of sight) toward visual targets at known locations (*e.g.,* ±10° to the right or left of straight ahead); subjects who have lost vision cannot be expected to do this with precision. Although these studies have provided informative, qualitative records of the eye movements shown by blind eyes, their lack of quantification has precluded any direct comparison with the eye movements of normal subjects.

Fortunately, there is a method to make reliable binocular measurements of eye movements in those who are unable to look at visual stimuli: the magnetic field and search coil technique [31]. This technique uses a silastic annulus, in which loops of fine wire are embedded, which can be calibrated on a protractor-gimbal device prior to placement on the subject’s topically anesthetized eye. This method has been used in eye movement research for over 30 years, and is regarded as the most reliable and sensitive method to measure 3-D eye rotations [2], [32], [33].

We studied a group of individuals with variable severity of visual loss and compared their gaze stability and saccades with a group of healthy control subjects. We were primarily interested in the case of monocular visual loss, in which the normal eye serves as a control. In such individuals, the abnormal eye often drifts about its visual target, predominantly in the vertical plane; this is called the Heimann-Bielschowsky phenomenon (HBP) [19], [22]–[28] and stands in contrast with gaze stability in normal subjects, in whom vertical eye movements are conjugate, especially during vertical saccades [34]. Upward saccades depend on bilateral projections from saccade-generating burst neurons in the rostral interstitial nucleus of the medial longitudinal fasciculus (RIMLF) in the midbrain to motoneurons innervating elevator muscles of each eye [35], [36], an anatomical arrangement that normally guarantees tight conjugacy of movements. Downward saccades, in contrast, depend on ipsilateral projections from burst neurons to motoneurons, an organization that might not guarantee such tight conjugacy [37]. We asked whether vertical saccades were disjunctive in individuals with monocular visual loss who had developed monocular gaze instability with disjunctive drifts. Since the pulse of innervation (eye velocity signal) is projected directly from saccadic burst neurons to ocular motoneurons, it seemed possible that vertical saccades would be more conjugate than eye drifts during attempted fixation. We also had the opportunity to measure voluntary saccades made to somatosensory cues in individuals with binocular visual loss occurring either during adulthood or dating from infancy.

We found that monocular visual loss affected monocular gaze stability more than vertical saccades, pointing to the importance of binocular visual inputs in optimizing the performance of the gaze-holding network. We also found that remarkably accurate saccades could be made to somatosensory cues following binocular loss of vision in adulthood, but not if blindness was from infancy. Some data from one individual with monocular visual loss and another with blindness since birth have been previously reported [19].

## Subjects and Methods

### Ethics Statement

All subjects gave informed written consent in accordance with Cleveland Veterans Affairs Medical Center Institutional Review Board and the Declaration of Helsinki. The Cleveland Veterans Affairs Medical Center Institutional Review Board specifically approved this study.

We studied six subjects with monocular visual loss (hereafter referred to as patients, P1-6) and two subjects with binocular visual loss (P7-8); demographic data, etiology, and severity of their visual loss are summarized in Table 1. These patients did not have pre-existing ocular motor abnormalities and were not taking any medications that could cause nystagmus. We compared their gaze stability with a group of 20 healthy control subjects (CS), all with visual acuity better than 20/30 in each eye; there were 8 females, with ages ranging 25–72 years, and a median of 55 years.

**Table 1:** Demographic and Clinical Information.

Patient/Age/Sex	Diagnosis/Duration	Visual Acuity OD – OS	Fixation abnormalities in the eye with impaired vision
P1/39/M	Trauma to left eye at age 9 years;aphakic until age 35 years, whenlens implanted; subsequentoscillopsia OS	20/20–20/25; No stereopsis	Drifts, without nystagmus, both vertically and horizontally, of left eye during binocular or right-eye viewing; similar drifts of right eye with left eye viewing
P2/49/M	Right eye injury as child witheventual recovery. Oscillopsianoted following minor headtrauma at age 44 years	20/25–20/20; No stereopsis	Drifts, without nystagmus, mainly vertically, and more marked in right eye; frequent square-wave jerks
P3/49/M	Congenital cataract in left eyewith severe visual loss sinceinfancy, not treated; retinal tearin right eye age 48 yrs	20/30– NLP; Monocularvision	Continuous up and right-beating nystagmus, with superimposed slow vertical drifts of eye position; right-beating nystagmus in his right eye
P4/29/F	Hypothalamic astrocytomaresected at age 16 yrs;bitemporal hemianopia andcentral vision loss in right eye(right optic neuropathy); vertical oscillopsia OD since age 27	20/200–20/20; Nostereopsis	High-frequency (pendular) oscillations, greater vertically, in right eye, with superimposed vertical drifts; upbeat nystagmus in left eye
P5/80/M	Wet ARMD OD; dry ARMD OS	Count fingers at 4 feet–20/25	Vertical drifts with superimposed upbeat nystagmus when right eye fixates; upbeat nystagmus when left eye fixates; frequent square-wave jerks
P6/83/M	Dry ARMD OD; Wet ARMD OS	20/25– Count fingers at2 feet	Drifts, with left-beating nystagmus, more evident in left eye; frequent square-wave jerks
P7/25/F	Leber’s congenital amaurosis;no visual memories	NLP	Continuous nystagmus with horizontal and vertical components, and a drifting null point
P8/60/M	Methanol poisoning at age 57 causingsevere bilateral optic neuropathies	Hand motion at 2 feet inupper field; no form vision	Continuous up- and left-beating nystagmus; frequent square-wave jerks

ARMD: age-related macular degeneration; OD: right eye; OS: left eye; NLP: no light perception.

Patients and control subjects sat with the head firmly stabilized by supports attached to a chair. We measured eye movements using the magnetic field and search coil technique [31], [32]. We pre-calibrated eye search coils prior to placement on subjects’ eyes; thus, calibration was not dependent on subjects’ ability to point their line of sight at visual targets. Either 2-D (horizontal and vertical) or 3-D eye and head rotations were measured, as previously described [33], [38]. The standard deviation (SD) of the noise of our system was ±0.02° and its linear range was ±30°. Fixation stability was tested as subjects attempted to view a small red target subtending 0.1° at the central position on a tangent screen at viewing distance of 1.4 m in an otherwise dark room. They viewed, in turn, with their right eye, left eye, or both. The duration of experimental runs during which subjects attempted to fix upon the visual target ranged from 10–60 seconds. Verbal encouragement was provided to subjects to sustain steady fixation of the small target during the test period, allowing occasional blinks. In addition, we tested visually-guided saccades as the visual target (a projected laser spot) jumped to eccentric horizontal or vertical positions in the range 5–20°; target jumps occurred at 1 Hz, but were unpredictable in direction. In the patient with acquired bilateral blindness who retained voluntary control of his eye movements (P8), saccades were tested as he attempted to shift his eyes between the thumbs of his outstretched hands, which were positioned about 20° eccentric to center.

Horizontal and vertical eye and head position were obtained from coil signals following analog filtering (pass-band 0–150 Hz) and digitization at 500 Hz with 16-bit precision [33]. Eye velocity signals were computed as previously described [33], [39]. Data were analyzed interactively using programs written in MatLab (Mathworks). For each fixation session, we first inspected the record to determine qualitatively the nature of the fixation disturbance – whether due to slow drifts, nystagmus, or saccadic intrusions. We then measured the standard deviation (SD) of eye position based on a minimum of 5,000 points. We also computed median eye speed as an estimate of absolute retinal image motion, this being mainly a measure of ocular drifts in the presence of nystagmus [40]. These measurements were made in the horizontal, vertical, and torsional directions (except for P2, 7 and 8, for whom only horizontal and vertical measurements were possible). We compared each set of data from our patients with visual impairment with corresponding pooled data from control subjects using the Mann-Whitney rank sum test, since the distributions of data were not normal. Statistical significance was set at p<0.05, unless otherwise stated. Rather than compute bivariate contour ellipses [41], we compared each subject’s eye drifts in each direction and in this way we tested predictions of our hypothesis: (1) loss of binocular visual cues due to monocular visual impairment will affect gaze stability in both eyes, but mainly in the eye deprived of vision, and (2) eye movements dependent on projections to the ocular motoneurons that by-pass the neural integrator, such as the upward saccadic pulse command, will produce more conjugate movements than the eye drifts that occur during attempted fixation.

## Results

### Visual Fixation Stability in Healthy Control Subjects

**Figure 1:**
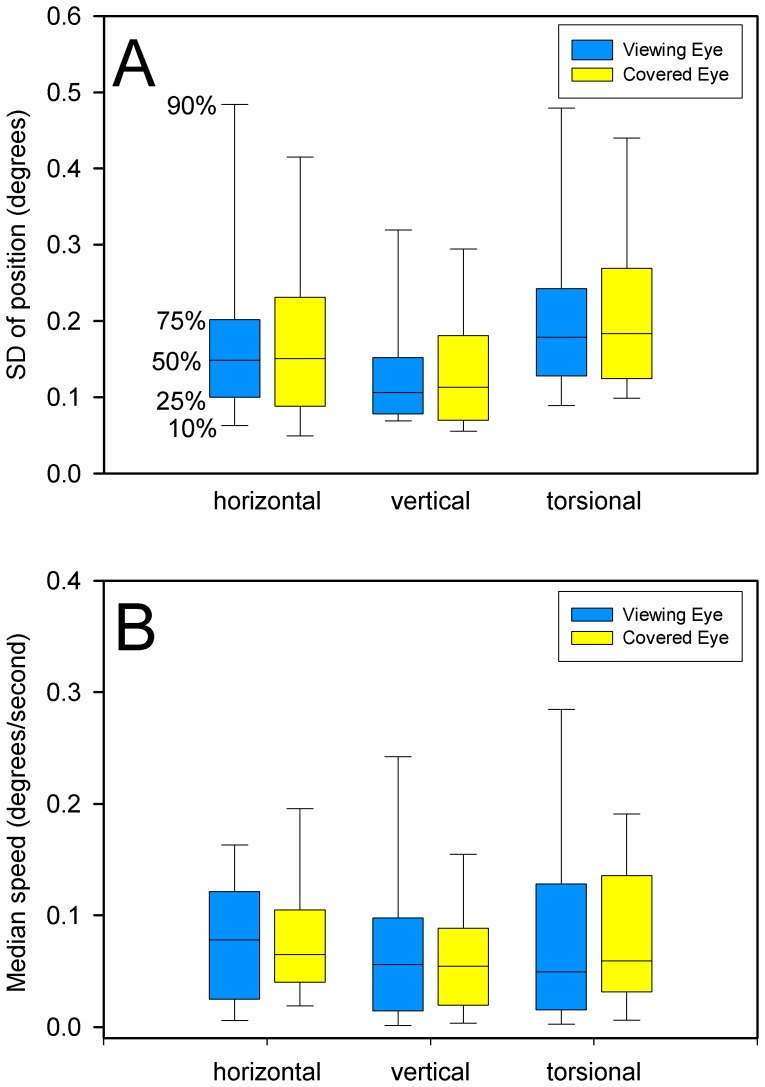
Summary of the stability of gaze in 20 healthy control subjects as they fixed upon a small visual target with one eye while their other eye was covered. Panel A shows the distribution of measurements of standard deviation (SD) of eye position; the percentile values on these box plots, and in subsequent figures, are indicated (50% is the median). Panel B summarizes median speed of eye drifts during monocular fixation. For the group of subjects, there was no significant difference in the SD of eye position or median speed between the viewing and covered eyes.

Our first question was whether our healthy CS showed relative gaze instability (evident as a greater SD of position) of the eye under cover during monocular viewing. We found no significant difference between their two eyes in any plane (Figure 1); median SD of gaze was <0.18° in all directions. Furthermore, median eye speed was <0.1°/s in all directions, and was similar in the viewing and covered eyes. These data are summarized in Figure 1. Accordingly, we pooled the data from both eyes from the 20 CS.

### Binocular Visual Fixation with Monocular Visual Loss

**Figure 2:**
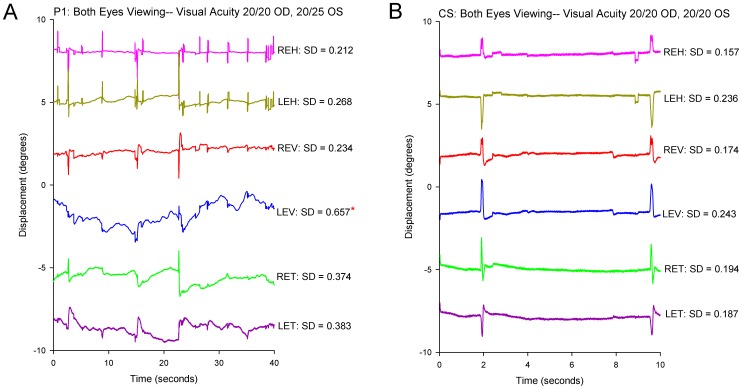
Representative records comparing binocular fixation behavior in P1 with monocular visual impairment versus a control subject. The scales are similar to allow direct comparison between P1 (A) and the control subject (B). In this and similar subsequent time plots of eye movements, positive values indicate eye rotations to the right, upward, or clockwise from the subject’s viewpoint. At the top, visual acuity of each eye is stated. At the right, the SD of eye position (in degrees) is specified for each directional component of their eye movements. REH: right eye horizontal; LEH left eye horizontal; REV: right eye vertical; LEV: left eye vertical; RET: right eye torsional; LET: left eye torsional. Note the increased instability of gaze in P1’s left eye, especially in the vertical plane (LEV). The asterisk indicates that the SD value is significantly larger (p<0.05) than pooled data from normal subjects.

**Figure 3:**
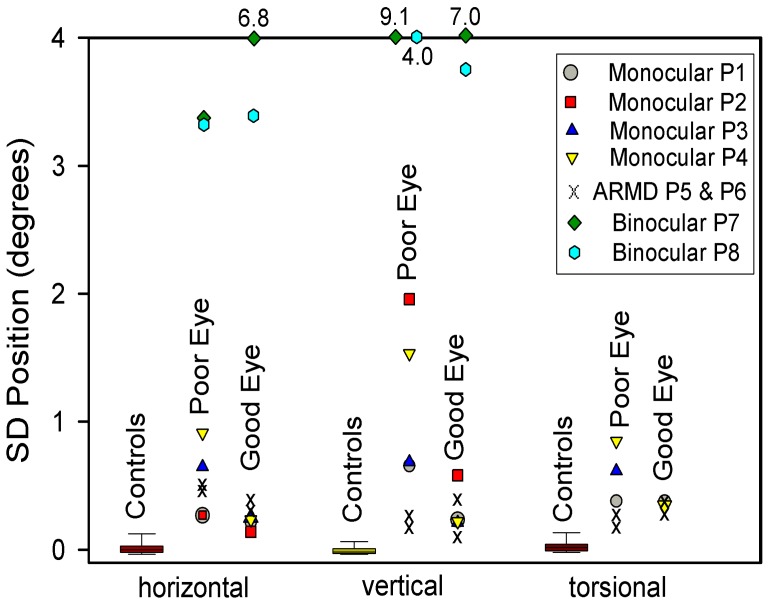
Summary of measurements of gaze stability, expressed as SD of eye position, for 20 control subjects (box plots) and for individual patients studied for each directional component. Note how especially vertical gaze has greater SD values, indicating greater instability, in the poor eye (lower visual acuity) of patients with monocular visual loss compared with control subjects. Also note how P7 and P8, with binocular visual loss, have much greater SD values (most unstable gaze) compared with either control subjects or patients with monocular visual loss. Outlier values are stated at the top of the plot.

Our next question was whether gaze was less stable in the eye with visual loss than our CS. First we considered the four patients (P1-4) whose visual loss dated from childhood or adolescence. All showed greater instability in the affected eye; an example is shown in Figure 2A and compared with a representative record from a CS in Figure 2B. The difference between these four patients with early onset of monocular visual loss and CS was evident from SD of gaze position, which is summarized in Figure 3. The increased SD was especially prominent in the vertical plane; P1-4 all showed a significant increase in SD for vertical position in the affected eye, and P3 and 4 also showed a significant increase for torsional position. An additional trend, evident in Figure 3, is that the eye with better vision was also more unstable (larger SD of position) than CS, although this was only significant for P2.

**Figure 4:**
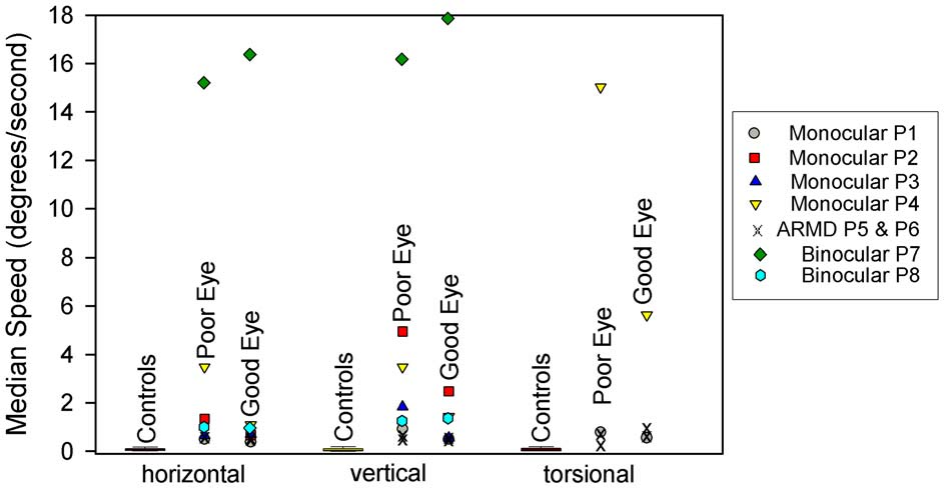
Summary of measurements of gaze stability, expressed as median eye speed, for 20 control subjects (box plots) and for individual patients studied for each directional component. For patients with monocular visual loss, SD values of eye speed were greater in the eyes with poorer vision (less so for P5 and P6 with late onset of visual loss due to ARMD). The fastest eye-drift speeds were shown by P7, who had been blind since birth.

Measurements of median speed gave generally similar results (Figure 4), with significantly faster drifts (measured as median speed) in all directions, but especially vertically and torsionally, in the eye with impaired vision.

**Figure 5:**
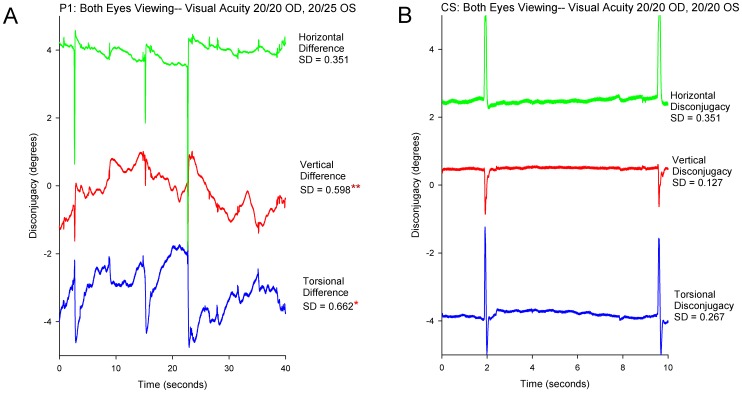
Representative records comparing the disconjugacy of gaze for each directional component for P1 with monocular visual impairment versus a control subject. (A): Record of P1. (B): Record of control subject. Values shown at right of each plot are SD of the difference between right and left eye position for each directional component. One asterisk indicates that the SD value is significantly larger (p<0.05) than pooled data from normal subjects; two asterisks indicate p<0.01. See caption to Figure 2 for further details.

A finding that follows from these inter-ocular differences is that their eye movements during attempted fixation were disjunctive – much more so than for the CS; Figure 5 compares the difference between the right and left eyes (disjunctive movements) of P1 and a CS. Comparison of the SD of the difference between the position of each eye in each direction showed significant differences for P1-4 in the vertical direction, and also in the torsional direction when this was measured binocularly (P1 and P4). When we calculated the ratio of median speed of vertical drifts in the bad/good eyes this was 1.8 for P1, 2.0 for P2, 3.3 for P3, and 2.4 for P4.

When we compared SD of the position of the affected eye of the two individuals with visual loss in later life due to ARMD (P5-6–shown as x’s in Figure 3), there were only small differences compared with CS, and little disconjugacy of drifts. However, the median speed of drift of either eye was significantly greater in each direction from controls. The ratio of median speed of vertical drifts in the bad/good eyes was 1.1 for P5 and 1.0 for P6. Thus, the differences in gaze stability between the two eyes for P5 and P6 were smaller than for P1-4 with early onset of monocular vision loss. These findings in P5 and P6 are consistent with a prior study of fixation stability in a large group of ARMD patients [30]. One possible reason to account for this difference is that ARMD patients have predominantly central visual loss, but retain the ability to detect binocular cues in their periphery of vision.

### Monocular Visual Fixation with Monocular Visual Loss

**Figure 6:**
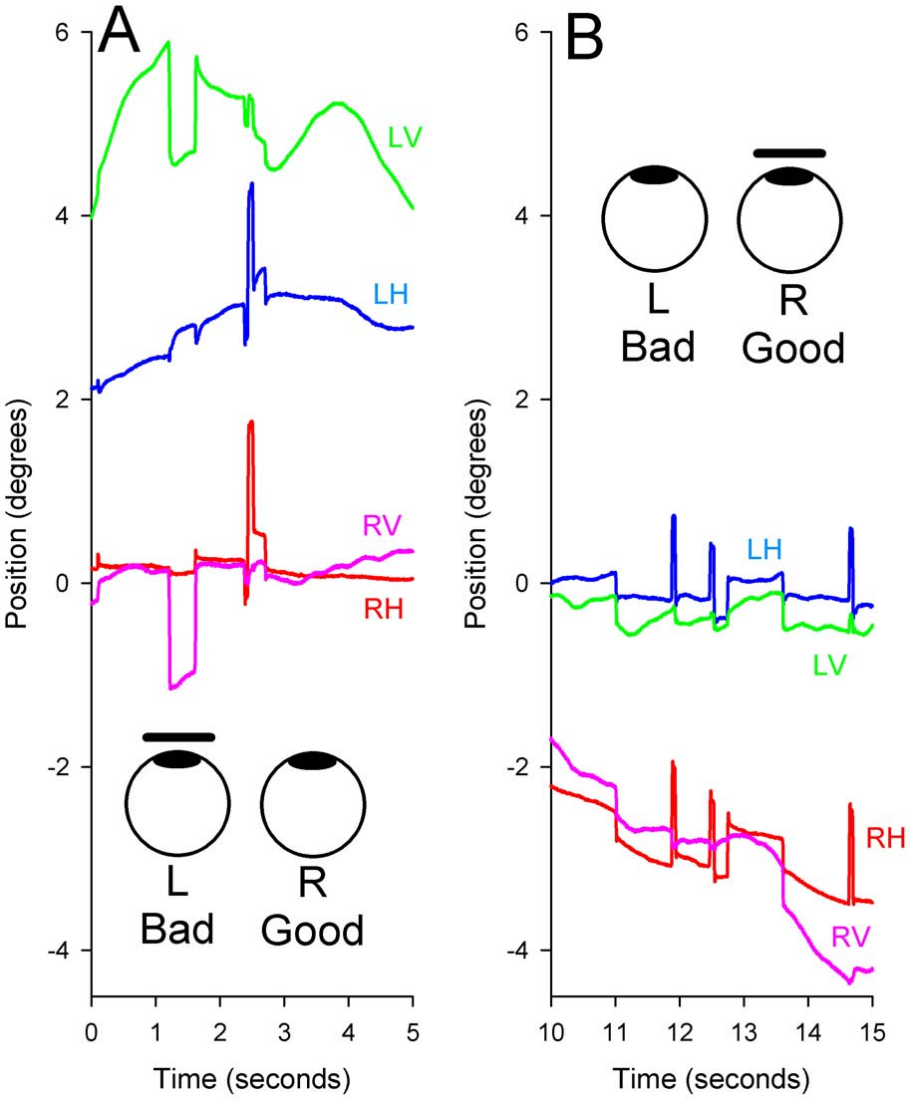
Representative record from P1 comparing the effects of monocular viewing with either eye. A: Viewing with the eye with better visual acuity (see cartoon at bottom). B: Viewing with the eye with poorer visual acuity (see cartoon at top). During fixation with the good, right eye (A), drifts are evident in the covered left eye, especially vertically. When the bad eye attempts to fixate the small visual target (B), gaze becomes more stable for that eye, but the good eye under cover shows increased drifts.

Since it had been suggested by Graefe that gaze stability may be improved when patients with monocular visual impairment attempt to view with their affected eye [22], we studied the effects of occluding the “good” and impaired eyes in turn. We confirmed an improvement of gaze stability in the eye with poorer vision in 4/6 patients when it fixed (and the other eye was occluded); an example from P1 is shown in Figure 6A. However, an unexpected finding in 3/6 patients was that, when the good eye was occluded, it became more unstable (SD of position increased); an example from P1 is shown in Figure 6B. Similar results have been reported for patients with amblyopia [41].

### Upward Saccades with Monocular Visual Loss

**Figure 7:**
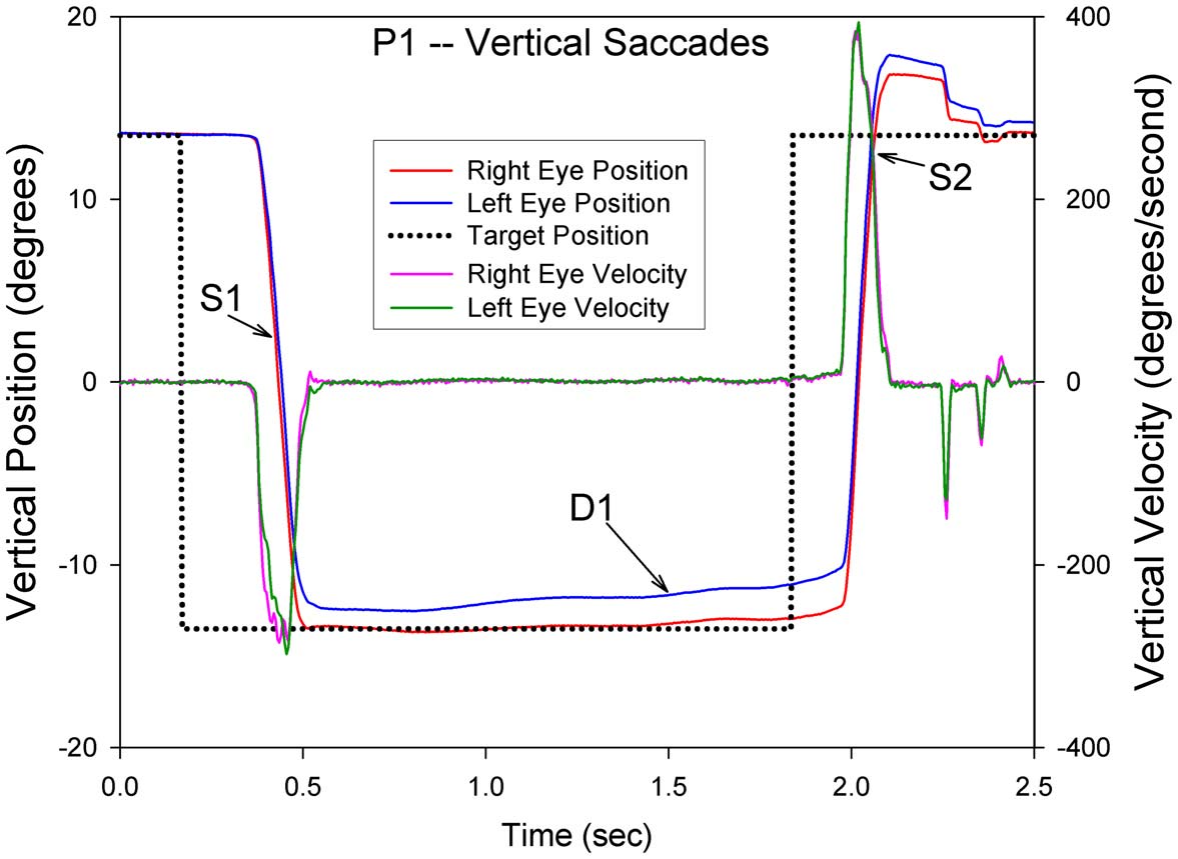
Representative record of vertical saccades made by P1. The left axis displays eye and target position and the right axis displays eye velocity. Target position is indicated by the dotted line. The first, downward saccade (S1) is mildly disjunctive (right eye, red trace, moves farther), although peak velocities of the two eyes are similar. Subsequently, the left eye (blue trace) drifts away from the target (D1). The second, upward saccade (S2), which has a small overshoot, starts with the eyes at different positions, but the change in eye position is similar and the velocity profiles are very conjugate (overlapping records).

**Figure 8:**
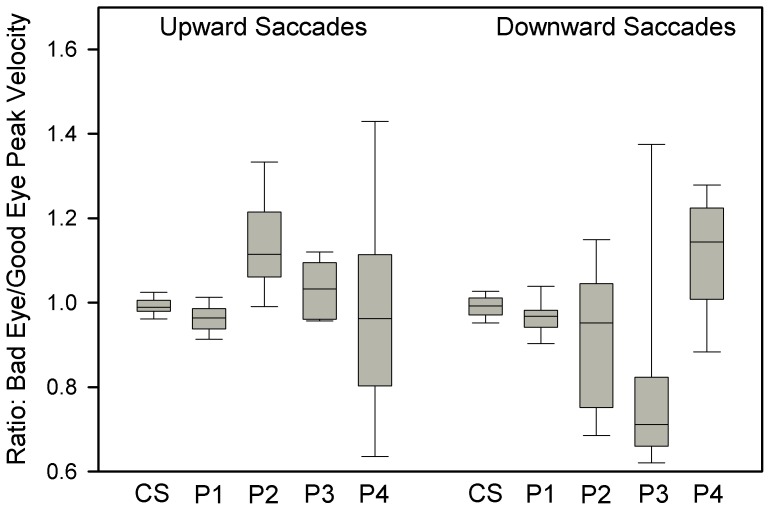
Summary of measurements of ratio of peak velocity of bad eye/peak velocity of good eye for upward and downward saccades of the group of control subjects (CS) and P1-4, who had monocular loss of vision early in life. Box plot conventions are similar to Figure 1. Perfectly conjugate saccades would have a ratio of 1.0. Upward saccades made by the patients are generally more conjugate than downward saccades.

To test the second part of our hypothesis – that eye movements due to signals passing directly to ocular motoneurons will be less affected by monocular loss of visual inputs – we measured the conjugacy of vertical saccades in P1-4, and compared them with control subjects. Our approach was to interactively measure the peak velocity of each eye and compute the ratio of bad eye peak velocity to good eye peak velocity. Representative vertical saccades from P1 are shown in Figure 7 and the results for CS and P1-4 are summarized in Figure 8. Median peak velocity ratios of upward saccades were within 1% of the conjugacy for CS in both directions. For upward saccades, median peak velocity ratio was 0.96 for P1, 1.11 for P2, 1.03 for P3, and 0.96 for P4. Thus, upward saccades deviated from conjugacy (1.0) by up to 11%. Downward saccades were less conjugate, deviating by up to 29%. It is also evident in Figure 8 that P1-4 showed more variance of peak velocity ratios than CS. Nonetheless, the peak velocity ratio values for vertical saccades were much smaller than the ratios of median speeds of vertical drifts of each of P1-4, which deviated from conjugacy by 180–330%. Thus, upward saccades were more conjugate than downward saccades, and both types of vertical saccades were much more conjugate than the drifts occurring during attempted fixation.

### Binocular Visual Loss

**Figure 9:**
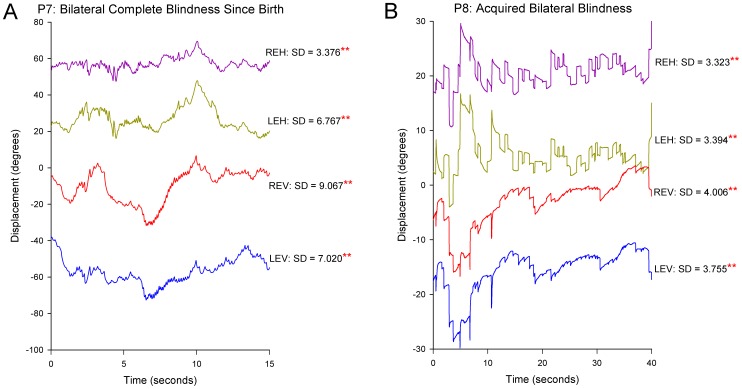
Representative records of gaze instability in two patients with bilateral visual loss. (A) Gaze instability shown by P7, who had been blind since birth. Conventions are similar to Figure 2. The records show continuous eye drifts and nystagmus that changes direction, implying a “wandering null” or variable set point of the gaze-holding mechanism. (B) Gaze instability shown by P8, who had lost vision binocularly 3 years previously due to methanol poisoning. Horizontal gaze is disrupted by bidirectional drifts and saccadic intrusions; vertical gaze is disrupted by downbeat nystagmus. Double asterisks indicate SD values significantly different (p<0.01) from control subjects.

We found that the SD of gaze position was much larger in our two binocularly blind patients than in CS or in those with monocular visual loss (Figure 3). Median eye speed was greatest in P7, who had been blind since birth (Figure 4); she showed a wandering null point (Figure 9A). P8, who had lost most binocular vision three years previously, showed eye drifts with speeds similar to that of monocularly affected patients (Figure 9B).

**Figure 10:**
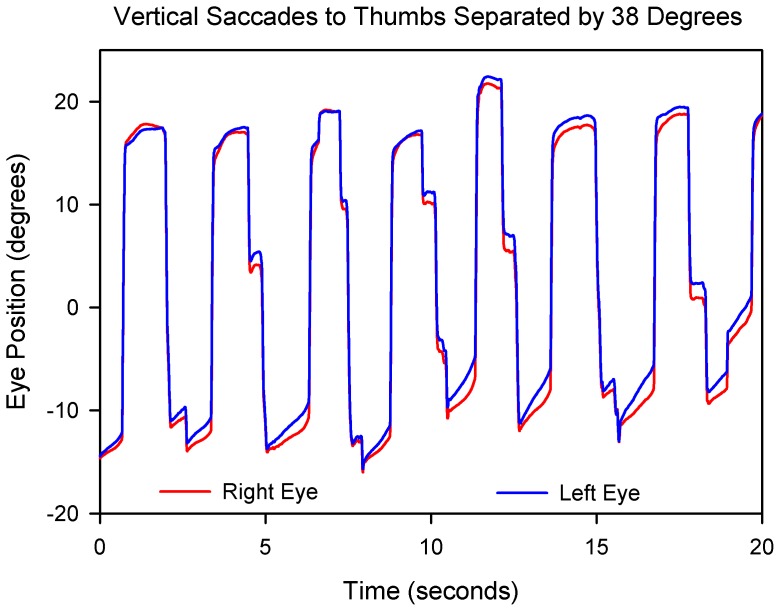
Vertical saccades made by P8 to his (unseen) thumbs located at positions above and below his eye level, subtending a visual arc measured to be 38°. The saccades are generally accurate and conjugate, despite upward drifts of gaze.

Finally, we asked how accurate eye movements could be in the case of P8, who had lost almost all of his vision 3 years previously. He retained some visual perceptions of space and could walk from his house to his garage. Using his thumbs as a somatosensory cue, he was able to make quite accurate saccades (Figure 10). In contrast, P7, who had been blind since birth, was unable to make voluntary saccades, although her nystagmus showed frequent quick phases (Figure 9A), implying that her brainstem saccade-generating mechanism was at least partly preserved.

## Discussion

We set out to study the effects of monocular visual loss on binocular stability of gaze by making reliable eye movement recordings and comparing gaze stability in these patients with a group of healthy control subjects. Prior studies have demonstrated that normal subjects can hold both eyes steady, even when one eye is occluded [42]. However, patching a normal subject’s eye for several days can cause misalignment of the visual axes (strabismus), which can take several hours to resolve [43].

We tested the hypothesis that the instability of gaze occurring with visual loss is a consequence mainly of loss of calibration of the neural network (integrator) for eye movements, which has been shown to contain component neurons that have monocular firing preference [15]. Two predictions of this hypothesis are that (1) loss of binocular visual cues, due to monocular impairment of vision, will affect gaze stability in both eyes, but mainly in the eye deprived of vision, and (2) eye movements dependent on projections to the ocular motoneurons that by-pass the neural integrator, such as the saccadic pulse command, will produce more conjugate movements than the eye drifts that occur during attempted fixation. Specifically, since individual burst neurons in the RIMLF project to motoneurons supplying elevator muscles of both eyes, due to axon collaterals [35], [36], this circuitry would be expected to generate upward saccades that are consistently more conjugate than the eye drifts that occur during attempted fixation. Our findings are generally supportive of this hypothesis. Vertical eye drifts occurring in eyes with visual loss were more disjunctive – by an order of magnitude – than vertical saccades. It is worthwhile noting that even normal subjects show some disconjugacy of vertical saccades when they shift gaze between two targets lying at different distances [44]. An additional test of our hypothesis would be to compare the conjugacy of vertical eye movements in response to pitch head rotation and vertical optokinetic stimuli. Our current study focused on head-fixed visual fixation and saccades, and we have only limited data from a prior study of vertical optokinetic responses that included Patient 1 [45] and demonstrated no gain asymmetry. Thus, systematic measurements of the conjugacy of vertical vestibular and optokinetic response in subjects with monocular visual impairment seems justified.

Our findings raise several issues for discussion. First, why is gaze stability in the eye with impaired vision mainly in the vertical plane (HBP)? Second, why is gaze stability of the eye with better vision impaired compared with control subjects, especially when it is covered? Third, why does it take time for these instabilities to develop? Fourth, why does HBP with oscillopsia sometimes persist despite recovery to relatively normal vision?

Vergence provides a robust, on-line mechanism to precisely align the eyes in the horizontal plane and, in Patient 1, was preserved, even though he lacked stereopsis. However, vertical vergence mechanisms show more limited range of movement and flexibility, although they are amenable to plastic-adaptive responses to changed visual demands, such as wearing a base-up prism before one eye [46]. However, our patients were unable to use every-day disparity cues to improve gaze stability in their eye with impaired vision, even when visual acuity was near normal following restoration of vision (P1 and P2).

The finding that gaze stability sometimes improved in the poorer eye when it was required to fix (by covering the good eye) was part of the original report of HBP [22]. But why should gaze stability of the eye with better vision decrease when it was covered while the poor eye was viewing (Figure 6)? Similar behavior has been previously reported in amblyopic individuals [41]. The combined behavior suggests that fixation stability of each eye is governed by a common neural network, and we propose that this is the “neural integrator” for eye movements [12]. It is well established that most premotor signals for eye movements (such as saccades) are velocity coded, but that the final eye movement command must specify eye position, or else the eye would drift back to its center position due to elastic restoring forces in the orbit. As noted in the Introduction, this neural integrator depends upon a distributed network of brainstem nuclei and the cerebellum. Although conventionally conceived as a network that guarantees conjugate gaze position, recent studies have emphasized the importance of constituent units with monocular preference [15]. If the neural integrator does guarantee the position of each eye, it needs to receive visual information from each eye to tune the balance of neurons such that the eyes move together. We propose that when lacking such inputs, the calibration of monocular units in the neural integrator deteriorates, leading to HBP. Bilateral loss of vision causes an even more severe breakdown in conjugate gaze holding, with continuous drifts of the eyes in ever-changing directions. This drifting null phenomenon (Figure 9) has also been reported following experimental cerebellectomy, which severely impairs the neural integrator function [47]. Interestingly, gabapentin has been reported to suppress the HBP [48]; this drug also suppresses forms of acquired pendular nystagmus that have been attributed to abnormalities (instability) of the neural integrator [49].

Third, our studies of bilaterally blind patients stress the importance of the duration and age-of-onset of visual loss. Thus, while our patient who was blind since birth due to Leber’s congenital amaurosis was unable to make accurate saccades, our patient who sustained almost complete vision loss at age 57 years was still able to make accurate saccades to proprioceptive targets three years later (Figure 10). It will be interesting to study how well voluntary ocular motor control returns in patients with Leber’s congenital amaurosis following vision restoration with gene therapy [50].

Finally, one might ask why the HBP persists despite relatively normal vision (P1 and P2) and oscillopsia. Development of oscillopsia after restoration of vision in an eye with HBP has been described by other groups of researchers [27]. It seems paradoxical that patients with HBP can perceive visual motion, but cannot use it to prevent the eye from drifting. Future studies to evaluate binocular tests of motion vision, such as deriving visual structure from motion, might provide insights into HBP, which is probably under-diagnosed, and which remains somewhat mysterious over a century after its original description.
